# Early Life Events and Maturation of the Dentate Gyrus: Implications for Neurons and Glial Cells

**DOI:** 10.3390/ijms23084261

**Published:** 2022-04-12

**Authors:** Viktor Aniol, Anna Manolova, Natalia Gulyaeva

**Affiliations:** 1 Institute of Higher Nervous Activity and Neurophysiology, Russian Academy of Sciences, 117485 Moscow, Russia; aniviktor@ihna.ru (V.A.); anna.manolova@ihna.ru (A.M.); 2 Research and Clinical Center for Neuropsychiatry of Moscow Healthcare Department, 115419 Moscow, Russia

**Keywords:** early life stress, hippocampus, dentate gyrus, maturation, neurogenesis, neuron, microglia, astrocyte, neuroinflammation, ontogenesis

## Abstract

The dentate gyrus (DG), an important part of the hippocampus, plays a significant role in learning, memory, and emotional behavior. Factors potentially influencing normal development of neurons and glial cells in the DG during its maturation can exert long-lasting effects on brain functions. Early life stress may modify maturation of the DG and induce lifelong alterations in its structure and functioning, underlying brain pathologies in adults. In this paper, maturation of neurons and glial cells (microglia and astrocytes) and the effects of early life events on maturation processes in the DG have been comprehensively reviewed. Early postnatal interventions affecting the DG eventually result in an altered number of granule neurons in the DG, ectopic location of neurons and changes in adult neurogenesis. Adverse events in early life provoke proinflammatory changes in hippocampal glia at cellular and molecular levels immediately after stress exposure. Later, the cellular changes may disappear, though alterations in gene expression pattern persist. Additional stressful events later in life contribute to manifestation of glial changes and behavioral deficits. Alterations in the maturation of neuronal and glial cells induced by early life stress are interdependent and influence the development of neural nets, thus predisposing the brain to the development of cognitive and psychiatric disorders.

## 1. Introduction

The dentate gyrus (DG) is an important part of the hippocampus, the main entrance to hippocampal formation playing an important role in learning, memory, and control of emotional behavior. DG retains high potential for neural plasticity, and its prolonged maturation in the postnatal period and lifelong neurogenesis in the subgranular zone contribute to an immense capability of DG to transform. Therefore, factors potentially influencing normal development of neurons and glial cells in the DG during its maturation can exert long-lasting effects on brain functions. In this review, the effects of early life events on DG maturation are comprehensively reviewed.

## 2. The Structure of the DG in the Adult Brain

Similar to other hippocampal structures, DG has three distinct histological layers: molecular, granular, and polymorphic (also called the hilus) [1,2,3]. The most superficial molecular layer is composed mainly of afferent axons and branching dendrites of granule neurons located in the next layer. The afferent glutamatergic pathways from the entorhinal cortex (perforant path) terminate on the dendrites of granule cells in the outer two-thirds of the molecular layer, whereas axons from hilar neurons of DG terminate at the inner one-third of the layer [4]. The granule neurons of the DG demonstrate rather similar morphology, with a main dendrite protruding through the granule cell layer and branching in the molecular layer. However, the molecular expression profile of granule cells may vary by cell age and position in the granule neuron layer (e.g., Prox1-, calretinin-, or calbindin- expressing cells) [5]. The inner border of the granule neuron layer is the place of adult neurogenesis and contains neural stem cells (NSCs) in a specialized neurogenic niche [6,7,8]. The polymorphic layer of the DG (the hilus), located most deeply and enclosed within the branches of the DG, contains small populations of excitatory and inhibitory interneurons that modulate the signaling pathways from the DG [4].

## 3. Pre- and Postnatal Development of the DG

An outstanding feature of DG development, making it different from other cortical structures, is the emergence of the secondary neurogenic niche, not associated with the ventricle wall, due to migration of neural precursors from the ventricular zone medially to the site of prospective DG. DG also differs from other hippocampal structures by its delayed maturation. In comparison with the Ammon’s horn located dorsally, the DG begins its development later and, at least in rodents, terminates it already after birth [9]. Moreover, this prolonged postnatal maturation of the DG during the first weeks of life continues in adulthood in the form of adult hippocampal neurogenesis [10].

The DG begins its development as a part of the hippocampal primordium, which makes up the medial ventricle wall of the telencephalon. In rats, at the beginning of the third week of embryonic development, the proliferation area of future dentate granule cells (the primary dentate neuroepithelium) begins to grow and separate from the neighboring Ammon’s horn progenitor area. On embryonic day (ED) 17, the time of spreading subicular and Ammon’s horn structures along the medial ventricle wall, the DG primordium is still present as a small group of cells. This primary dentate neuroepithelium locates around the dentate notch, a ventricular indentation between the Ammonic neuroepithelium dorsally and the fimbrial glioepithelium ventrally [9]. At this stage, Wnt signaling determines the expression of the Lef1 transcription factor in the cells of the dentate neuroepithelium. This is crucial for proper patterning of this area since the absence of Lef1 leads to a phenotype with no dentate neuroepithelium and no granule neurons [11].

Starting from this time point, dividing cells originating from the dentate neuroepithelium leave the site of their birth and begin their migration medially towards the nascent hippocampal fissure, building a clearly visible dentate migration stream on ED19 [9]. During this migration, the cells remain mitotically active. This migration is guided by the long processes of radial glial cells protruding from the dentate neuroepithelium [12]. On their way, this migration stream contributes to some other transient secondary neurogenic niches, e.g., in the hilus and molecular layer [5,13]. This stage in rodents begins prenatally, though it mostly takes place during the first two weeks of postnatal life [9]. During this stage, cells express transcription factors essential for regulation of neuronal differentiation, e.g., Prox1 and Neurod1 [14,15]. Reelin, an extracellular signaling protein released by Cajal–Retzius neurons, is another important factor guiding DG morphogenesis. These neurons originate from the cortical hem, a structure directly adjacent to the dentate neuroepithelium [5,13]. Differentiation of Cajal–Retzius cells and reelin signaling in this region are critically dependent on different transcription factors, including Foxg1 and Emx2, which limit Cajal–Retzius cell production to the cortical hem, and Lhx5, which is important for their proper migration and differentiation [16,17]. In turn, reelin signaling is important for proper laminar organization of the DG and for correct establishment of afferent input from the perforant path [18,19].

Neurons produced locally from stem-like GFAP+ radial glia cells give rise primarily to principal glutamatergic projection neurons, the granule cells of the DG [5,12,20,21,22]. GABAergic interneurons, quite similarly to the situation with neocortical development, tangentially migrate to the DG from distant areas of the lateral and medial ganglionic eminences [23].

At birth, the DG is still immature and completes its development during the first weeks of postnatal life, with a majority of granule neurons comprising the adult rodent DG being generated during the first postnatal week [24,25]. In humans, the granule cell layer of the DG is also immature at birth and continues to grow, increasing by roughly a half during the first 3 months of postnatal life [26,27].

The rate of neurogenesis within the subgranular zone (SGZ) gradually decreases with aging [25]. In rats, the number of proliferating cells in the DG decreases 10 times (from approximately 6000 to 600 cells/day per DG) between the first and seventh month. In the DG, no clear border exists between embryonic and postnatal neurogenesis despite some molecular features of the progenitor cells. Thus, during prenatal development, most cells in the DG originate from GFAP+ progenitors, but soon after birth these progenitor cells start expressing the basic lipid-binding protein (BLBP) as well, thus shifting to a GFAP/BLBP double-positive phenotype during adult neurogenesis [28]. The majority of cells generated in the SGZ both pre- and postnatally become granular neurons [29]. During differentiation lasting for roughly a month, these cells demonstrate changes in protein expression typical for consecutive stages of neuronal maturation, similarly to what is observed during embryonic neurogenesis: from markers of undifferentiated stem-like and progenitor cells (nestin and PSA-NCAM) to neuroblast markers (doublecortin, dcx) and mature granular neurons (calbindin, NeuN); after several divisions the stem-like GFAP+ cell becomes a post-mitotic astrocyte [30,31]. Aging generally does not affect the fate of new cells generated in the DG, one-half of them being eliminated by roughly 1 month after their birth and 70–80% of remaining, integrating into the granule cell layer as calbindin+ granule neurons [24]. However, a decrease in the number of symmetric divisions following the activation of stem-like cells may result in accumulation of post-mitotic astrocytes in the neurogenic niche [31].

Given that proliferation of neural precursor cells and migration of their progeny to their final positions in the granule cell layer continues after birth, it seems reasonable to assume that early postnatal interventions affecting the DG eventually result in: (a) an altered number of granule neurons in the DG; (b) ectopic location of neurons; (c) and changes in adult neurogenesis. Below, we will discuss these consequences reported in different models of early life stress.

## 4. Effects of Early Life Events on Neuronal Structure of the DG

Hippocampal development may be particularly vulnerable to various insults during embryogenesis and soon after the birth since this region remains highly neurogenic postnatally due to prolonged maturation of the DG. Long-term changes in the basal rate of adult neurogenesis are believed to be associated with significant cognitive and behavioral abnormalities. Chemical or genetic suppression of adult hippocampal neurogenesis impairs animal performance in certain hippocampus-dependent learning and memory tasks [32,33,34]. A decrease in neurogenesis induced by systemic inflammation was reported to be associated with anxiety- and depression-like behaviors [35,36,37,38].

### 4.1. Inflammation

Intrauterine exposure to inflammatory stimuli such as lipopolysaccharide (LPS) leads to white-matter damage and neuronal injury [39,40], as well as a reduced rate of neurogenesis in the hippocampal SGZ during the first weeks of postnatal development, resulting in decreased granule cell density in adulthood. Prenatal inflammation also results in the accumulation of ectopic hilar granule neurons, suggesting aberrant migration of newly born granule cells [41]. The basal level of neurogenesis in adulthood after prenatal LPS exposure is reduced if LPS is administered during early embryogenesis (before the beginning of the 3rd gestational week) [42] or remains unaffected if LPS exposure occurs later [41]. Similar results were reported by Järlestedt et al. [43] and Smith et al. [44] after early postnatal exposition to inflammatory stimuli in mice. Systemic LPS administration during the first postnatal days did not affect the total proliferation rate in the DG at different time points (from several days to more than 2 months), while proliferation of neuroblasts reduced. However, opposing observations were also reported: Pang et al. [45] found that early life systemic LPS injection (on PD 3) resulted in increased proliferation of precursor cells in the DG later in adulthood. Despite certain differences in experimental approaches, there is still no clear explanation of the inconsistency in the data reported; genetic background (different animal strains were used) was supposed to be the most probable influencing factor in this case. Though outside of the current review scope, it should be noted that in the adult hippocampus the rate of neurogenesis is usually decreased in response to inflammatory stimuli (see [46] for review).

The long-term fate of newly born cells affected by neonatal LPS exposure was followed up by Järlestedt et al. [43]. Neonatal inflammation did not affect continuing survival of neurons that were already post-mitotic at the time of the impact. However, the viability of cells born under inflammatory conditions was reduced in the long-term perspective, and the total number of newly generated neurons and astrocytes was reduced in adulthood.

The effects of early life LPS on the viability of young neurons and adult neurogenesis in the DG can be exerted through inflammatory cytokines, since their level is essentially increased after systemic LPS administration, while the IL-1 antagonist IL-1Ra prevents detrimental effects of LPS on DG neurogenesis [47,48]. Similarly, neonatal injection of IL-1β suppresses the effect on the proliferation rate of adult Tbr2+ progenitors in the DG [49].

Another possible mechanism affecting the DG is oxidative stress, a phenomenon often associated with inflammation. Indeed, systemic administration of LPS to neonatal rats is followed by rapid elevation of brain superoxide dismutase, and long-term effects may be mediated by Nrf2 and PGC-1α transcription factors, phosphorylated Akt, and elevated acetylation of histone 4 [50]. Three months after early life LPS administration, the level of gp91-phox/NOX2 subunit of NADPH oxidase 2 protein is still increased in hilar astrocytes, while the superoxide level is elevated in granule neurons judged by dihydroethidium staining. Oxidative stress and neuroinflammation may be linked through the P2X7 receptor involved in both cascades [51].

### 4.2. Early Life Stress

Common early stress models in rodents are based on limited nesting and bedding material or temporary maternal separation during the first postnatal weeks, when the DG is still forming, and stressful events are more likely to induce persistent impairments as compared to stress during adulthood [52]. Early stress directly enhances the proliferation and differentiation of immature NeurD1-positive cells in the dentate migration stream. However, long-term survival of these cells and, respectively, the size of DG are reduced in adult animals. Survival of adult-born neurons in the DG is also affected by early stress, but mainly in males, which also correlates with performance in cognitive tests [53]. The rapid stimulating effect of early stress on DG neurogenesis during the first postnatal days may be determined by decreased repressive H3K9 histone demethylation at the brain-derived neurotrophic factor (Bdnf) gene IV promoter along with enhanced levels of Bdnf gene expression. Suppressed neurogenesis later in adulthood is accompanied by opposite changes in Bdnf gene regulation [54]. These results suggest that early life stress, first enhancing the neurogenesis in the immature DG, leads to a subsequent slow depletion of the stem-cell pool in the adult DG [52].

### 4.3. Hormones

Final steps of postnatal DG development run in parallel with maturation of the endocrine control. It is no surprise that changes in the levels of hormones may seriously influence the structure of the DG. Normally, the cells of the developing DG are influenced by systemic testosterone after its conversion to estradiol by aromatase, an enzyme expressed in the DG during the first postnatal week. Transient expression of estrogen receptors also occurs during this period [55].

In the DG, neonatal testosterone promotes sex differences in cell genesis. Male rodents have approximately twice as many proliferating cells as females during the first postnatal week [56,57,58]. Early life administration of androgens to females enhances proliferation and survival of new neurons in the developing DG of neonatal females and males lacking a functional androgen receptor [59]. This correlates with the performance of animals in hippocampus-dependent tasks [60].

Progesterone receptors are transiently expressed on Cajal–Retzius cells in the molecular layer of the DG during the first weeks of postnatal life, peaking between PD7 and PD10, and their activation is important for the establishment of proper hippocampal circuitry [61,62]. A blockade of progesterone receptors during the first postnatal days induces accumulation of reelin in the DG and subsequent impairments in episodic memory [19].

### 4.4. Hypoxia

Hypoxia experienced during the first days of postnatal life can decrease the rate of adult neurogenesis, according to the number of doublecortin+ cells in the DG. This effect was dependent on the inflammatory cascade since early administration of IL-1Ra restored a normal neurogenesis rate [47]. Interestingly, early life hypoxia seems to have no effect on the number of neurons in the adult DG. However, hypoxic episodes experienced in early life may trigger epileptogenesis, which may manifest later in adulthood due to changes in AMPA-receptor composition, making it more permeable to divalent cations [63].

### 4.5. Seizures

Epileptogenesis is one of the most intriguing examples of long-term plasticity. Many manifestations of this aberrant plasticity are observed in the Ammon’s horn and DG, including cell loss, changes in adult neurogenesis, synaptic reorganization and long-term changes in the expression of ionic channels and receptors [64]. However, neonatal seizures are quite different from those occurring in the adult brain and they differently affect neuronal circuits. Seizures experienced during the first two postnatal weeks are not accompanied by essential neuronal loss in the DG, which is confirmed in many models, including lithium-pilocarpine [65], kainate [66] and fluorthyl seizures [67]. Neonatal seizures result in lasting changes in glutamate receptor composition and expression of ionic channels, but generally they do not alter the course of postnatal neurogenesis [63,68,69].

## 5. Maturation of Microglial and Astroglial Cells in the DG

Glial cells are important players in the process of brain maturation. They are involved in axon guidance, neurite growth, synaptic pruning, and apoptosis [70,71,72], thus regulating synaptic function, plasticity, and circuit formation throughout brain development [73,74,75,76]. Importantly, immature and mature glial cells perform different functions in the brain. Disturbances during early maturation of glia may result in long-term consequences affecting the adult period.

### 5.1. Postnatal Maturation of Astroglia

The first postnatal week is accompanied by active astrocyte proliferation [77]. It is also a period of active blood-brain barrier development with a prominent involvement of astrocytes [78,79]. At this time, astrocytes express thrombospondins (TSP1 and TSP2), key molecules inducing synapse formation. The expression of trombospondins is reduced by the third postnatal week [80], together with a decrease in the neuronal potential to generate synapses [81]. Glutamate receptors and transporters (EAAT-1 and GLT-1) are also expressed by astrocytes from the first postnatal week [82,83]. Astrocytes are considered to become mature around the end of the third postnatal week in coordination with synaptogenesis, which also takes place at this time. Morel et al. showed that on PD14-PD26, two interdependent events occur: excitatory synapse ensheathment and maturation of protoplasmic astrocytes and their processes in the neocortex [84]. Thus, trombospondins together with other molecules secreted by astrocytes such as glypicans (reviewed in [85]) are necessary for proper synapse formation during postnatal maturation of excitatory neuronal pathways.

Interestingly, interactions between astrocytes mature in parallel with the processes mentioned above. The expression of Connexin43 (Cx43), a marker of inter-astrocytic gap junctions, starts as early as PD1. However, immunoreactive staining of Cx43 on PD1–PD5 reveals fibrous elements and only by PD15 the staining becomes punctate similar to that seen in adult brain [86]. Territory occupied by an astrocyte changes during its maturation. During the first postnatal week, the astrocytic processes have filopodial morphology and intercept the territory of the neighboring astrocytes. By PD14, the overlapping is reduced by the pruning of some processes and boundary-like structures become distinguishable between distinct cells. These boundaries are clearly seen by PD21 [87]. Since these events coincide with synapse formation, factors disturbing normal astrocyte development have deleterious consequences on the neural network organization.

There is evidence that astrocytes are sexually dimorphic in early ontogenesis [88]. Sexual dimorphism in the pattern of GFAP expression at the early stages of ontogenesis was first shown in 1990s [89,90]. In the hippocampus and hypothalamus, the state of astrocytes depends on sex hormones and, therefore, the forming of neuronal nets has specific sex-related features.

### 5.2. Microglia Maturation in Early Ontogenesis

Microglia becomes mature earlier than astrocytes, around the end of the second postnatal week. By this time, it already displays a ramified morphology and gene expression pattern similar to that of adult microglia [91,92]. Moreover, different stages of the microglial maturation can be discriminated according to gene expression profile [92]. At the first stage (so called early microglia), microglial functions are mostly related to cellular phagocytosis. This stage lasts approximately from ED8.5 to ED14. This is also the period of microglial colonization of the brain. Next, the “pre-microglia stage” begins on ED14 and lasts until the end of the first postnatal week. During this period, microglia has a transitional phenotype and, besides phagocytosis, it also supports the axonal dynamics (by expressing DAP12 [93]), neuronal survival by expressing IGF1 (at the PD5 in the cortex [94]) and induces developmental neuronal apoptosis (at the PD1 in the hippocampus [95]). The next is a transitional stage preceding adult microglia and lasting for the second week of postnatal ontogenesis. At this stage, microglia begin to participate in the neuronal net formation by synaptic pruning [74,96,97]. Noteworthy, the timing of microglia development depends on the brain structure; for example, in the preoptic area, microglia participate in the synaptic patterning as early as PD2 [98]. From PD14, microglial cells express Tmem119 at the adult level and this is considered to be the marker of mature stage, together with the ramified morphology [92]. At the earlier and adult stages, microglial cells also make a contribution to myelination by expressing IGF-1 [99,100]. Another important marker of microglia maturation may be PU.1, a transcription factor activating gene expression during myeloid cell development [101] and expressed in microglia during the second and third weeks of postnatal development [102]. The paper by Lenz and Nelson [98] presents a detailed comprehensive review of microglial maturation staging.

The above data suggest that dysfunction at a certain stage of microglial development may perturb proper neuronal network formation, resulting in behavioral abnormalities and increased vulnerability to neuropsychiatric disorders.

### 5.3. Involvement of Microglia in Neurogenesis in the DG during Ontogenesis

Adult neurogenesis in the DG is accompanied by apoptosis of excessive, constantly generated immature precursor cells [103], thus necessitating proper utilization of apoptotic bodies by resident ramified microglia [104]. Normally, no activation of microglia is observed under moderate inflammatory conditions, i.e., changes of microglial shape from ramified to ameboid as well as phagocytosis of cellular remnants into phagolysosomes of the cell body. Apoptotic bodies are phagocytized by separate thin microglial processes without affecting the microglial body shape or inducing classical inflammatory activation molecular markers such as CD11b (αM-integrin) and CD68 (macrosialin) [105]. Despite the absence of morphological and molecular signs of activation, the phagocytic activity of microglia is high, allowing complete elimination of apoptotic bodies within 1–2 h [104]. Microglia also promotes survival and neuronal differentiation of maturing neuroblasts in the DG by secretion of BDNF and insulin-like growth factor-1 (IGF-1) [106,107,108]. During aging, microglial cells participate in maintaining physiological level of neurogenesis through fractalkine/CX3CR1 signaling pathway [109]. This pathway may provide a mechanism linking neuronal activity with the population of maturing neuroblasts maintained by microglial cells, which thus gain a key role in DG functioning [110].

The effects of microglial cells on neuronal maturation in the DG is dependent on their functional state [111]. The classic pathway of microglial activation is accompanied by increased production of proinflammatory cytokines IL-1, IL-6, and TNF-α, which previously have been shown to decrease the survival of young neurons [112,113]. On the contrary, an alternative way of microglial activation realized in some cases results in synthesis and secretion of Il-10 and prostaglandin E2, as well as BDNF and glial-derived neurotrophic factor (GDNF), which contribute to the survival of new neurons [114]. Interestingly, a regional heterogeneity of microglial populations exists, depending on their ability to support adult neurogenesis [108]. In addition to a prominent effect on neurogenesis, activated microglia may influence the properties of developing neurons in the DG by changing the structure of dendritic spines and enhancing the inhibitory inputs on newly generated synapses [115].

## 6. Responses of Microglial and Astroglial Cells to Aversive Events during Early Ontogenesis

It is well-established that glial cells possess great heterogeneity in different brain structures, including expression patterns and morphological features [116,117,118]. Considering developmental differences in hippocampal regions, the DG and Cornu Ammonis (CA), we further review the effects of aversive early life events considering these hippocampal parts. Since biochemical approaches do not provide the necessary spatial resolution to discriminate substructures inside the hippocampus, we complement them with histochemical data on glial cells state.

### 6.1. Immediate Effects of Aversive Events in Early Ontogenesis on Glial Cells

There are not many studies concerning immediate effects of stress in early postnatal ontogenesis on the state of glial cells in the hippocampus. Osborne et al. demonstrated an increase in the IL-1β mRNA level in the hippocampus 24 h after *E. coli* (ATCC 1547) injection on PD4. Yet, no signs of microglial activation were revealed in either the CA1 and CA3 subfields or in the DG [119]. Subcutaneous LPS injection on PD3 and PD5 resulted in an increase in GFAP+ but not Iba-1+ immunostaining optical density in the SGZ on PD8 [120]. Saavedra et al. showed an increase in the percent of activated microglia in the CA3 and DG 24 h after systemic LPS administration on PD14. It was accompanied by an increase in IL-1β level 90 min after LPS injection [121]. A decrease in the GFAP+ cells’ number in the CA3 and DG was revealed 24 h after systemic LPS injection on PD14; a 2-fold increase in the soma size of GFAP+ cells was evident in the DG only [121]. Daily maternal separation during the 2 first postnatal weeks did not increase the IL-1β level, though it resulted in a rise of the percent of activated microglia in both the CA3 and DG [121]. A decrease in astrocyte cell population density was revealed in both the CA3 and DG, though the number of astrocyte processes per cell was diminished only in the CA3 region [121]. Another study of maternal separation during PD10 [122], instead of PD 14 in [121], demonstrated an increase in the Iba-1+ immunostaining optical density in the hippocampus, while no changes in the GFAP+ immunostaining optical density were detected. A study of maternal separation during the 2 first postnatal weeks in mice also showed an increase in the microglial cell density population in the whole hippocampus [102]. This increase was accompanied by changes in the gene expression pattern showing significant dysregulation of 58 genes involved in the cell cycle and apoptosis (e.g., Casp8), pro-inflammatory activation (e.g., Il-1α, Il-1r1), cell migration (e.g., Cx3cr1) and phagocytosis (e.g., C1q, Itgam/CD11b). The results of the promoter analysis in the hippocampus suggest that maternal separation alters the microglial gene expression profile on PD14 by modifying the activity of the Creb1, SP1, and RelA (a subunit of the NFKB transcription factor) [102].

Some authors report even a decrease in the Iba-1 staining optical density, which may be attributed to reduction of the Iba-1 staining intensity or to the cell population density decrease. Unfortunately, it is virtually impossible to differentiate between scenarios taking place judging only by the immunostaining optical density, though this is the simplest and fastest way to get results. Hoeijmakers et al. showed that limiting the nesting and bedding material from PD2 to PD9 resulted in an increase in IL-1β expression in the hippocampus and simultaneously in a decrease in the Iba-1 staining optical density in the hilus of the DG on PD9 [123]. The optical density of GFAP immunostaining, a marker of astrocytes, was also decreased in the stratum lacunosum-moleculare of the hippocampus but not in other structures, including the DG. Early life stress induced neither changes in the morphological features of hippocampal astrocytes nor the mRNA expression of astrocytic markers such as Aldh1l1, Gfap, Vimentin, Aqp4, Fasn, Glast, Glut1, and GluS [124].

Thus, the majority of reports considering the immediate effects of early life stress on brain tissue demonstrate moderate neuroinflammation in the hippocampus manifested primarily as an elevation in interleukin levels, usually but not necessarily, accompanied by morphologically evident microglial activation. In contrast, astrocytes demonstrate rather a decrease in cell number or GFAP expression within the first day after the onset of an early life adverse event.

### 6.2. Delayed Effects of Early Aversive Events on Glial Cells

The long-term effects of early disturbances in the microglial functioning may be explained with a phenomenon called microglial priming. Exposure to glucocorticoids induces unspecific sensitization of microglial cells to subsequent pro-inflammatory stimuli [125,126,127]. It is noteworthy that another type of steroid hormone, estradiol, is also suggested to be a priming agent for microglia in early ontogenesis as well as the basis of microglial sexual dimorphism. The research groups of J.J. Watters and S.D. Bilbo showed that microglial cells should be sexually dimorphic to ensure normal ontogenesis [128,129,130]. Recently, several groups confirmed that adverse events during early ontogenesis have differential long-lasting effects on male and female subjects [131,132,133,134]. Some authors suggest that sex differences in microglia underlie distinctions in the occurrence of mental illnesses such as autism [135], Parkinson’s disease, and multiple sclerosis in men and women [136,137].

It is believed that subtle neuroinflammation induced by aversive early life events may persist during long periods. Banqueri et al. showed that maternal separation from PD1 to PD21 resulted in an increase in IL-6 mRNA expression in the hippocampus accompanied by increased microglial cell population density in the CA3 subfield but not in the CA1 or DG regions at the age of 3 months [138]. A decrease in the astrocytic cell population density was also evident in all hippocampal regions studied. In a study using the same animal model, Delpech et al. failed to show alterations in the microglial cell density and microglial cell surface area in the hippocampus on PD28, a week after the end of early postnatal stress [102]. Nevertheless, the gene expression pattern was influenced: 43 genes were dysregulated in the isolated hippocampal microglial cells. Promoter analysis indicated that the changes in the transcriptional activity of Creb1, Sp1, RelA, and PU.1 were responsible for 86% of these dysregulated genes [102]. In another animal model of early life stress, limiting the nesting and bedding material from PD2 to PD9, it was shown that the microglial cell population density only tended to increase in the molecular layer of the DG and stratum lacunosum-moleculare of the CA1 subfield in mice at the age of 4 months [123]. However, if the assessment of microglial cells was performed by staining CD68+ cells instead of Iba1+ cells, a significant increase in optical density was evident in both the DG and CA regions. In addition, there was a trend to increased CD11b mRNA expression and a significant decrease in IL-6 mRNA expression, though early life stress did not affect IL-1β and TNF-α mRNA expression in the hippocampus [123]. In the same experimental paradigm, no effect of early stress on the state of hippocampal astrocytes at the age of 4 months could be revealed, though a decrease in the astrocytic cell population density in the DG hilus was demonstrated at the age of 6 months [124]. None of the astrocyte-related genes (Aldh1l1, Gfap, Vimentin, Aqp4, Glast, Glut1, GluS) mRNA expression was influenced at both ages, while an increase in the Fasn mRNA expression in the hippocampus of stressed mice was shown at the age of 4 months only [124]. It is noteworthy that the expression of fatty acid synthase (encoded by Fasn) in the hippocampus was found only in astrocytes but not in neurons, and astrocytic lipid metabolism was shown to be critical for presynaptic terminals’ development in vivo [139].

Some authors report the absence of neuroinflammatory signs in the adult brain of rodents exposed to early life stress. Ganguly et al. reported no influence of maternal separation on microglial morphological features and cell-population density in the prefrontal cortex at the age of 4 months, but the authors did not assess levels of cytokines [140]. Single studies report even a decrease in the cell population density of microglia in response to aversive early life events. Recently, we showed such a decrease in the DG of females but not in the CA of females or in the male hippocampus on PD18 after systemic LPS injection on PD3 and PD5 [141]. Thus, although aversive events in early life may induce signs of neuroinflammation in the hippocampus, later this effect becomes quite moderate and sometimes cannot be seen without additional impact.

## 7. Effects of Early and Late Aversive Events Combination on Glial

Recently, hypotheses of two-hit etiology of mental diseases were suggested by several authors. The hypothesis was initially proposed for schizophrenia [142], but at present it is suggested for a number of other mental diseases potentially associated with early life stress [143]. Briefly, this hypothesis supposes a necessity of two impacts for disease onset. The first impact is aversive event(s) in early ontogenesis that primes glial cells and modifies their reaction to subsequent stressful events, the response becoming more proinflammatory. The second hit represents a variety of potential event(s) in adulthood, e.g., proinflammatory challenge, behavioral stress, even genetic vulnerability. From this perspective, the experimental design utilizing animal models of early life adverse events should always include an intact group (animals undisturbed until the end of the experiment) since even behavioral testing may interfere with proper interpretation of the results. The lack of this group may cause either misinterpretation or contradictory results.

Berkiks et al. reported that LPS injection on PD14 resulted in an increase in the number of astrocytes in the dorsal part of the hippocampus in male and in dorsal and ventral parts in female Wistar rats, as well as an increase in the microglial cell number in the ventral hippocampus of both males and females in adulthood [144]. However, all groups underwent behavioral testing, including a stressful forced-swimming test before sacrificing. In this case it is impossible to distinguish whether the reported effect is due to early life stress per se or is a result of a combination of early life and adult stress factors. Indeed, most studies designed to prove the double-hit hypothesis show statistically significant effects of interaction of early life stress and adult stress. Diz-Chaves et al. reported an increase in microglial and astroglial cell density in the DG only in response to the combination of prenatal stress and LPS injection at the age of 4 months [145]. Similarly, aging in combination with *E. coli* injection at PD4 led to a more pronounced increase in the expression of glial markers mRNA (CD11b, GFAP) in the hippocampus of 16-month-old Sprague-Dawley rats [146]. Saavedra et al. showed that combined maternal separation for the first two postnatal weeks and LPS administration on PD15 induced a more pronounced increase in the percent of activated microglia in the CA and DG, as well as a significant increase in the soma size of astrocytes in the DG as compared with the respective effects of each factor alone [121]. Interestingly, the elevation in expression of interleukin mRNA (IL-1β, IL-6, TNF-α) was reduced after the combination of two stress factors as compared with LPS injection alone.

Neonatal systemic LPS injection in combination with acute restraint stress in adulthood (but not LPS alone) induced a dramatic increase in IL-1β content in the hippocampus of male and female rats [147]. The acute restraint stress in adulthood alone induced only a moderate non-significant elevation of IL-1β. In this study, the content of TNF-α also increased in response to a combination of early life and adult stress but only in the hippocampus of male rats, while IL-6 content in the hippocampus of males and females did not change in response to any of the impacts [147].

In summary, adverse events in early life provoke proinflammatory glial changes at cellular and molecular levels immediately after early life stress exposure. Later, the changes at cellular level may disappear, though alterations in the gene expression pattern persist. Stressful events later in life, the second hit, contribute to manifestation of glial changes. A cross-talk between the astrocytes and microglia is very important when considering their negative effect on neural networks. However, we could not find studies aimed at revealing such interplay using a model of early life stress, as was done in the research performed by Lana et al. on the normal and aged hippocampus [148]. It is noteworthy that even the data reported separately for microglia or astrocytes clearly demonstrate that the differences in the functioning of glia primed by early life stress inevitably affect the functioning of neuronal networks.

## 8. Conclusions

**Figure 1:**
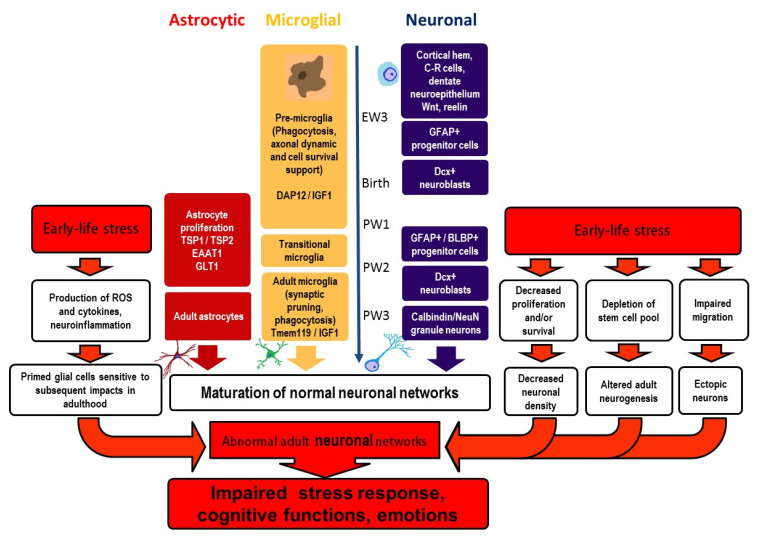
Maturation of neurons and glial cells in the dentate gyrus and effects of early life stress. Early life stress interferes with maturation of both neurons and glia in the DG, resulting in the development of abnormal neural networks in adulthood. These aberrant networks form the basis for perturbed stress response and the development of brain pathologies (cognitive and emotional disturbances), their manifest being potentiated by stressful experiences in later life. BLBP—basic lipid-binding protein, C–R cells—Cajal–Retzius cells, DAP12—DNAX activating protein of 12 kDa, Dcx—doublecortin, EAAT1—excitatory amino acid transporter-1, EW—embryonic week 3, GFAP—glial fibrillary acidic protein, GLT1—glutamate transporter-1, IGF-1—insuline-like growth factor-1, PW—postnatal weeks, Tmem119—transmembrane protein 119, TSP 1 and 2—trombospondines 1 and 2, ROS—reactive oxygen species.

The DG is a significant part of the hippocampus vitally involved in learning, memory, and emotions. Factors potentially influencing the normal development of neurons and glial cells in the DG during its maturation can exert continuing effects on brain functioning. Early life events may alter maturation of the DG and induce lifelong alterations in DG structure and function, these modifications underlying brain pathologies in adults. We have summarized the current knowledge on maturation of neurons and glial cells (microglia and astrocytes) in the DG and the effects of early life events on these maturation processes (Figure 1). Early postnatal interventions interfering with the DG maturation eventually result in an altered number of granule neurons in the DG and affect adult neurogenesis and neuronal migration, including ectopic location of neurons. Early adverse events provoke proinflammatory glial changes immediately after stress exposure at the cellular and molecular levels. Later cellular changes may disappear, though alterations in gene expression persevere. Additional stressful events in adulthood contribute to the manifestation of glial changes and behavioral disturbances. Modifications in the maturation of neuronal and glial cells induced by early life stress are interdependent and impact the development of neural networks, thus predisposing the brain to the development of cognitive and mind disorders.

## Data Availability

Not applicable.
